# Epidemic, Endemic, or Stewart–Bluefarb? When Several Forms of Kaposi Seem to Dispute Paternity

**DOI:** 10.1155/2020/6289285

**Published:** 2020-04-09

**Authors:** Hugues Adegbidi, Bérénice Dégboé, Fabrice Akpadjan, Nadège Agbessi-Mekoun, Christiane Koudoukpo, Alida Kouassi, Félix Atadokpede

**Affiliations:** ^1^Dermatology and Venereology Service, National University Hospital CNHU-HKM, University of Abomey-Calavi, Cotonou, Benin; ^2^Dermatology and Venereology Service, Department University Hospital of Parakou (CHUD-Parakou), University of Parakou, Parakou, Benin

## Abstract

The role of human herpes virus 8 (HHV8) is demonstrated in the occurrence of Kaposi's disease, but the role of cofactors is still hardly known. We report a case of Kaposi's disease which occurred 10 years after a local trauma in an HIV-positive patient from Central Africa. A 38-year-old female, from and living in Central Africa, consulted for angiomatous papulo-nodules associated with purple-colored macules and painful lymphoedema of the right leg and foot that had been developing for 6 months. She reported a history of posttraumatic lymphoedema of the affected limb as a result of a road accident that occurred ten years earlier. The mucous were healthy. There was no sign of systemic lesions. The diagnosis of Kaposi's disease was evoked with, in differential, a Stewart–Bluefarb syndrome-type of pseudo-Kaposi and an epidemic Kaposi disease. Retroviral serology was positive to HIV1 with a CD4 count of 600 cells/mm^3^. Histopathology of the lesions and duplex ultrasonography could not be performed. The rest of the biological assessment was without particularity. The diagnosis of epidemic Kaposi's disease associated with cofactors involved in endemic Kaposi's disease and Stewart–Bluefarb syndrome was retained. An antiretroviral treatment (emtricitabine, tenofovir, and efavirenz) allowed to obtain after 6 months a noticeable improvement of the lesions and a disappearance of the pain with however the persistence of a residual lymphoedema. This is a special case of Kaposi's disease that seems to involve several factors. The role of cofactors in Kaposi's disease remains to be elucidated.

## 1. Introduction

Kaposi's disease (KD), previously named Kaposi's sarcoma, was first described in 1872 by Moritz von Kaposi. It is characterized by cutaneous and/or visceral tumor lesions following after the proliferation of fusiform cells and dermal vessels. It is caused by an infection with HHV8 (human herpes virus 8) also called KSHV (Kaposi's sarcoma-associated herpes virus). Depending on the context, there are currently four forms of KD: classical, endemic, epidemic, and iatrogenic [1–3].

Since the advent of the HIV-AIDS (human immunodeficiency virus-acquired immunodeficiency syndrome) pandemic, it has experienced an epidemiological outbreak. The risk of developing KD is higher in areas where there is a high prevalence of HHV8 infection and HIV infection: Central, West, and South Africa [4–8].

Stewart–Bluefarb syndrome is a form of pseudo-Kaposi corresponding to reactional posttraumatic angiodysplasia. It is clinically and histologically similar to MK [9].

Although the role of HHV8 in KD is demonstrated in the occurrence of KD, cofactors remain poorly understood [1–3]. We report here a case of KD occurring after a 10-year-old vascular trauma on a patient from Central Africa with HIV infection.

## 2. Observation

**Figure 1:**
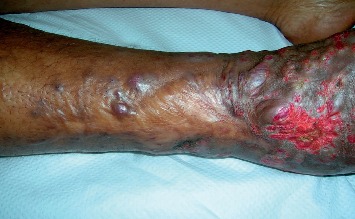
Kaposi's disease showing angiomatous papulo-nodules on lymphoedema of the right leg and foot.

A 38-year-old woman who is from and lives in Central Africa consulted for solid lesions and purple-colored patches on her right leg and foot that had been evolving for six months. The patient's interview revealed a notion of pain of the affected limb, an antecedent of trauma of the same limb in a road accident that had occurred ten years before and that resulted in a leg lymphoedema. The physical examination found the patient in good general condition, without fever. Locally, there were infiltrated purple macula associated with angiomatous papulo-nodules and lymphoedema of the right leg. The external part of the lower third of the same leg is the seat of an unsightly and atrophic scar and erosion of the external malleolus (Figure 1). At palpation of the pulse, there was no thrill or associated breath at auscultation. The mucous were unharmed. There was no sign of impairment of other organs. So, the diagnosis of endemic KD was evoked in view of the geographical origin of the patient, with a pseudo-Kaposi of Stewart–Bluefarb and an epidemic KD being differentially diagnosed. Hemogram revealed nothing particular. C-reactive protein was positive at 35  mg/L. The histopathology of the lesions and the ultrasonography of the affected limb have not been performed. HIV serology was positive to HIV1. The CD4 count was 600 cells/mm3. The diagnosis of epidemic KD on an anteriorly traumatized leg in a patient from Central Africa was retained.

**Figure 2:**
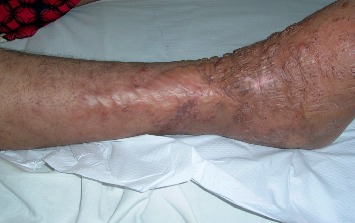
Kaposi's disease showing fewer papulo-nodules on residual lymphoedema of the right leg and foot after 6 months of antiretroviral treatment.

The patient was put on a combination of antiretrovirals including two nucleoside reverse transcriptase inhibitors (emtricitabine-tenofovir) and a non-nucleoside reverse transcriptase inhibitor (efavirenz). The development was marked by the complete disappearance of the pain and an important regression of the lesions after six months of treatment, with however the persistence of residual lymphoedema (Figure 2).

## 3. Discussion

Our clinical case poses a real diagnostic problem. Despite the positivity of retroviral serology, endemic KD and Stewart–Bluefarb syndrome could not be formally eliminated.

The responsibility of the HHV8 is no longer to be demonstrated in the occurrence of the KD. Anti-HHV8 antibodies are found in at least 95% of all forms of KD cases. However, prevalence and clinical forms differ according to geographic areas and underlying terrain [1–3, 10, 11]. The presence of HHV8 therefore appears to be necessary but not a sufficient condition for the onset of KD. Genetic, immune, and environmental cofactors were referred to [1, 2]. With the case of our patient, we also suggest local cofactors such as microtrauma or tissue remodeling in the stimulation of the virus and the proliferation of fusiform cells.

With the influence of the environment, endemic KD occurs in several forms: indolent cutaneous, acral aggressive, disseminated, and generalized lymphadenopathy in children with bad prognosis. The regions of high endemicity are Latin America, South Africa, and West and Central Africa, the region of origin of our patient. Seroprevalence to HHV8 is around 40–90% compared to 3–5% in the countries of Europe (north, south, and west), Asia, and North America [2, 11, 12]. Endemic KD affects adults as well as children. Its transmission is more probably horizontal (child-child) or vertical (mother-child and parents-children) [1, 3, 6–8]. It is diagnosed most often between 30 and 40 years with a marked predominance with males. However, in these areas there is no parallelism between the frequency of seroprevalence with HHV8 and that of endemic KD [4–6].

With the advent of HIV infection, the once rare KD has experienced a real outbreak. Its prevalence among HIV-positive patients varied between 15% and 29% with a clear male predominance during the 1980s. Its sexual transmission is demonstrated in homosexuals. Currently, its incidence has decreased considerably with the introduction of antiretroviral combinations (ARVs) [1–5].

KD associated with epidemic HIV is particularly aggressive with bilateral or profuse cutaneous involvement, mucosal, and systemic involvement all in the context of severe immunosuppression [1, 11, 12]. Indeed, the Th1-type cytokines secreted during HIV infection potentiate the activation of HHV8 resulting in an increase of viral load and higher risk of occurrence of KD. Also, the activating protein “Tat” which intervenes during the replication of HIV type 1 stimulates the secretion of proangiogenic chemokines, which will in turn induce the development of KD through the release of fibroblastic growth factors. This latter mechanism may account for the fact that KD is more common with HIV1 than with HIV2 infection [1, 3, 4, 11].

Immunosuppression plays a key role. For example, in areas with high seroprevalence of HIV and HHV8, the prevalence of epidemic KD is often higher. Central Africa's countries range among the most affected by this coinfection. In these countries, the prevalence of epidemic KD varies between 8% and 18% [1–3, 5, 6].

The positive retroviral serology in our patient was a strong argument in favor of an epidemic KD. However, the absence of severe immunodepression and the unilateral character also pointed to an endemic localized KD more than epidemiological factors, or a pseudo-Kaposi type Stewart–Bluefarb, due to previous trauma.

Stewart–Bluefarb pseudo-Kaposi is a rare condition that affects young adults between 20 and 30 years, with predominance in males. It is secondary to a reactive angiodysplasia affecting the blood vessels. Its pathogenesis is not clearly elucidated, but it suggests that any condition favoring the increase of venous pressure produces a retrograde blood flow that can stimulate, especially in fragile anatomical areas, the proliferation of endothelial and fibroblastic cells under the action of the vascular endothelium growth factor (VEGF). The contributing factors may be trauma, as is the case in our patient, an arteriovenous fistula during hemodialysis, vascular surgery, or a significant hormonal period such as puberty or pregnancy [9, 13].

The diagnosis of the Stewart–Bluefarb syndrome with Kaposi's disease is clinically and histologically difficult. The labeling of perivascular cells by CD34 antigen, the detection of HHV8 viral DNA, the presence of crystalloid intracytoplasmic inclusions, or the positivity of factor VIII R antigen were put forward as elements that enable the distinction between a pseudo-Kaposi and a KD [1, 14]. In its classic form, Stewart–Bluefarb syndrome occurs as purple, infiltrated, well-defined, firm, and sometimes sensitive plaques. Sometimes, it is purple-colored nodules associated with brown spots and localized oedema as is the case in our patient. The lesions are unilateral acral or hanging, of sudden appearance. The duplex ultrasound of the limb associated with arteriography reveals the arteriovenous fistula [9, 13, 14].

The treatment of epidemic KD is different from that of other forms. In other forms, immunosuppressive chemotherapy or radiotherapy is often used for the treatment of diffuse forms. In epidemic KD, combined ARV treatment is recommended [3, 10]. The action of ARVs is very probably indirect. By inhibiting the replication of HIV, they induce the restoration of the immune system, which results in the decrease of the HHV8 viral load and thus the partial or total regression of KD, thus confirming the opportunistic nature of this tumor [10]. Moreover, it is shown that early ARV treatment and limited forms of epidemic KD allow remission in at least 80% of patients [12]. Additional chemotherapy or radiotherapy may be necessary to better control of the tumor [1, 3].

Given the therapeutic and prognostic implications and subject to the exact date of the HIV infection, we have retained the diagnosis of epidemic KD precipitated by the cofactors implicated in the endemic KD and the Stewart–Bluefarb-type of pseudo-Kaposi. The evolution was favorable with an important regression of the tumor under ARV combination.

## 4. Conclusion

Our observation poses a diagnostic problem of a unilateral localized form of KD in an HIV seropositive patient living in a HHV8 seroprevalence zone. It has allowed us to draw attention to the fact that KD can be multifactorial and can lead to an intricate clinical picture. It could also involve for a given clinical form the combination of genetic, immunological, environmental, and local cofactors. Could KD, then, be a continuum in which the weight of one of the factors involved could predispose to a particular clinical form?
